# Protective effects of capillary artemisia polysaccharide on oxidative injury to the liver in rats with obstructive jaundice

**DOI:** 10.3892/etm.2012.666

**Published:** 2012-08-14

**Authors:** CHANG-SHENG HE, HONG-YI YUE, JIAN XU, FENG XUE, JIAN LIU, YUAN-YUAN LI, HONG-EN JING

**Affiliations:** 1 Department of Hepatobiliary Surgery, Jiaozhou Central Hospital of Qingdao, Qingdao, Shandong 266300;; 2 Department of Pediatrics, The First People’s Hospital of Baoding City, Hebei, Baoding 071000;; 3 Department of Hepatobiliary Surgery, Affiliated Hospital of Qinghai University, Xining, Qinghai 810001, P.R. China

**Keywords:** capillary artimesia, obstructive jaundice, oxidative damage, polysaccharide

## Abstract

Obstructive jaundice is a condition caused by blockage of the flow of bile out of the liver. This results in an overflow of bile and its by-products into the blood, and bile excretion from the body is incomplete. Untreated, obstructive jaundice can lead to serious infection that spreads to other parts of the body. We examined the protective effect of capillary artemisia polysaccharide on oxidative damage to the liver in growing rats with obstructive jaundice (OJ). Growing male Wistar rats (n=40, age 3–4 weeks) were randomly divided into four groups (n=10 in each group): normal control group, sham group, OJ group and OJ with capillary artimesia polysaccha-ride treatment group (study group). The rats of the OJ group and the study group were subjected to common bile dust ligation, while the sham group had the bile duct mobilized but not ligated. The rats of the study group recieved 5 ml/kg capillary artimesia polysaccharide (0.5 g/ml) by intraperitoneal (i.p.) injection once daily while the other groups were administered 5 ml/kg saline by i.p. injection. After 4 weeks, the rats were sacrificed to obtain liver weight and to compute the liver coefficient. Additional measures included liver homogenate malondialdehyde (MDA) and the activity levels of superoxide dismutase (SOD), glutathione peroxidase (GSH-Px) and catalase (CAT). The liver weight and liver coefficient of rats in the study group were lower than those in the OJ group and higher than those in the control and sham groups (P<0.05). Liver homogenate MDA content in the study group rats was lower than that in the OJ group and higher than that in the control and sham group (P<0.05). SOD, GSH-Px and CAT activities were higher in the study group rats than those in the OJ group and lower than those in other groups (P<0.05). Capillary artimesia capillary artemisia polysaccharide protects the liver from oxidative damage and improves antioxidant defense in growing rats with obstructive jaundice.

## Introduction

Obstructive jaundice (OJ) refers to a type of jaundice caused by mechanical obstruction of the extrahepatic and intrahepatic bile ducts due to tumor, stones, inflammation and other causes. Gallbladder tissue damage is a major pathological manifestation of OJ (1). According to the literature, obstruction of the biliary tract can cause reduced cholate in the intestinal tract and decreased ability to clear endotoxin, which can directly induce apoptosis of liver cells (2). Obstruction of the biliary tract also can impair electron transport chain function of liver mitochondria and promote oxygen free radical generation. Simultaneously, obstruction of the biliary tract reduces the activity of free radical scavenging enzymes in the liver, causing decreased free radical scavenging capacity and further induction of liver cell apoptosis.

Traditional Chinese medicine describes capillary artemisia as bitter, acrid and slightly cold, able to remove damp heat from the spleen, stomach, liver and gall bladder. It is considered an important drug for treating jaundice. Capillary artemisia impacts primary Qi of Shaoyang and affects the ascending vitality of liver. Its acridness can dispel stagnation of the liver and gallbladder (3). Studies show that capillary artemisia can protect the liver, relax the gallbladder and remove jaundice; this provides a pharmacological basis for treating liver disease with capillary artemisia (3–5). This study investigated the protective effects of capillary artemisia polysaccharide on oxidative injury to the liver using the common bile duct ligation method in growing rats.

## Materials and methods

### 

#### Experimental animals and grouping

Healthy male Wistar rats (n=40, age 3–4 weeks old, weight 85–95 g) were randomized into four groups (n=10 each): a normal control group, a sham operation group, a OJ group and a OJ + capillaris polysaccharide group or ‘study group’. The rats were kept in a quiet and ventilated environment with a room temperature of 22–26°C and relative humidity of 40–70%. All animals had free access to a normal chow diet and water during the experiment.

### Methods

#### Extraction of capillary artemisia polysaccharide

Dry powder of crushed capillary artemisia was weighed and added to water at a ratio of 1:20, then placed in a water bath at 80°C for 3 h. The sample was centrifuged to remove filter residues and harvest the supernatant. The supernatant was concentrated followed by addition of a 3-fold volume of anhydrous ethanol. The sample was centrifuged to obtain the sediment. Saline was added to prepare a 1% solution, and the sample was deproteinized with 3% trichloroacetic acid and freeze-dried to obtain crude polysaccharides (6). The phenol-sulfuric acid method was used to determine polysaccharide with glucose as the standard substance and a 721 spectrophotometer was used for colorimetric analysis (7). The extracted capillary artemisia polysaccharide was prepared as a 0.5 g/ml solution.

#### Preparation of a rat pup model of OJ

The rats were fasted for 12 and 4 h for food and drink, respectively. Rats were anesthetized with ketamine (20 mg/kg) by intraperitoneal (i.p.) injection, then cut along the mid-line of the upper abdomen to the hepatoduodenal ligament. The common bile duct was dissociated and ligated near the porta hepatis. Rats in the sham operation group underwent common bile duct dissociation but no ligation. The standard of a successful model preparation was that the OJ rat pup model had total bilirubin and direct bilirubin values (measured by blood sampling) that were 5-times higher than the normal control group two days after the procedure (8). Rat pups in the study group were administered 5 ml/kg capillary artemisia polysaccharide solution (0.5 g/ml) via once-daily i.p. injection; the other groups were given 5 ml/kg saline via once-daily i.p. injection. After 4 weeks, the animals were sacrificed and their livers were rapidly removed, washed with ice-cold saline, blotted dry and weighed. Liver weight was also expressed relative to grams body weight, as the ‘liver coefficient’. A sample (0.2 g) was taken from each liver, mixed with 0.2 mol/l phosphate buffer into 10% tissue homogenate and centrifuged at 3000 rpm/min. The supernatant was obtained, and xanthine oxidase superoxide was used to determine the activity level of superoxide dismutase (SOD). The NADPH coupling method was used to determine the activity of glutathione peroxidase (GSH-Px). Visible light was used to determine the activity of catalase (CAT) and thiobarbituric acid (TBA) was used to determine the content of malondialdehyde (MDA) (9).

#### Statistical analysis

SPSS 13.0 software was used for statistical analysis. The data are expressed as the means ± standard deviation (SD). Single factor variance analysis was used to compare the test results of each index across groups, followed with pairwise comparison between groups using the Student-Newman-Keuls test. The above analysis was performed as a two-sided test; the test level α was 0.05 with a P-value of <0.05 considered statistically significant.

## Results

### Liver weight and liver coefficient

**Figure 1:**
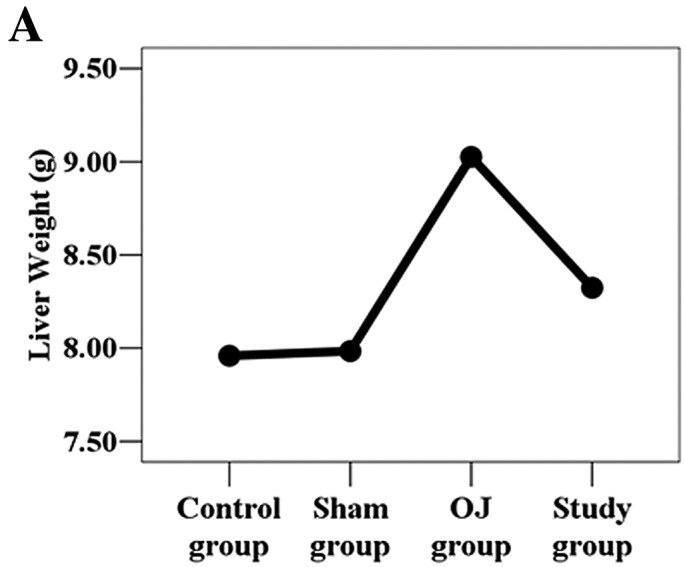

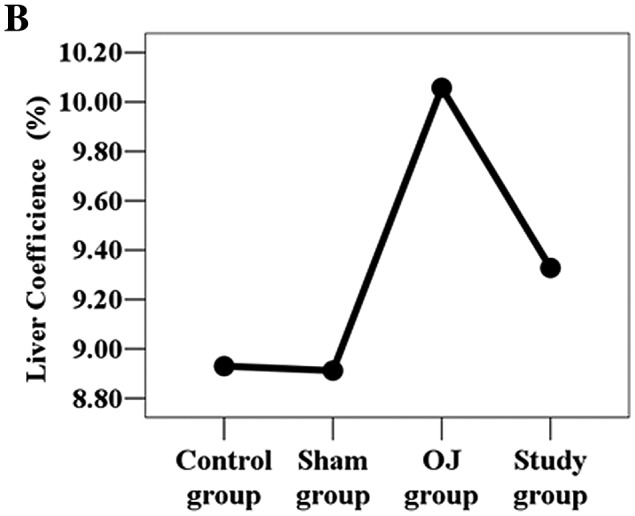
Changes in (A) liver weight and (B) liver coefficient among the groups.

The liver weight and liver coefficient in the normal control, sham operation, OJ and study groups were: 7.96±0.26 g, 8.93±0.40%; 7.98±0.27 g, 8.92±0.46%; 9.03±0.26 g, 10.06±0.38%; and 8.32±0.44 g, 9.34±0.57%, respectively. The liver weight and liver coefficient in the OJ model group were the highest followed by the study group, the normal control group and the sham operation group (Fig. 1). Liver weight and liver coefficient were statistically significant among the groups (P<0.05). Liver weight and liver coefficient for the study group were significantly reduced compared to the OJ model group, but still higher than the normal control group and the sham operation group (P<0.05).

### MDA content in the liver homogenate

**Figure 2:**
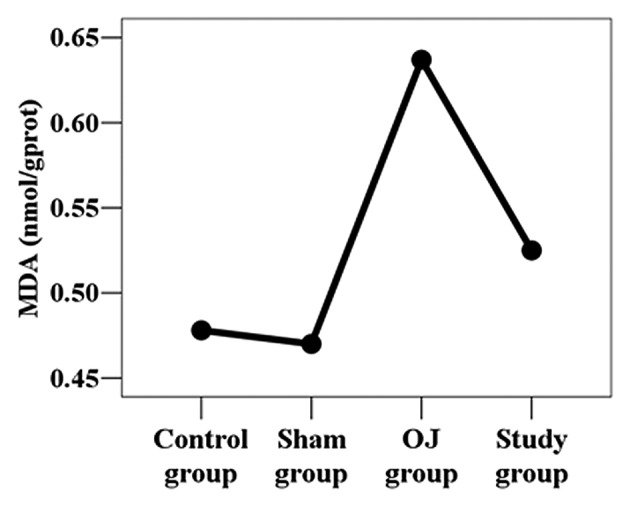
Changes in MDA content in liver homogenate among the groups.

MDA content in the liver homogenate was 0.48±0.01, 0.47±0.01, 0.64±0.05 and 0.53±0.04 nmol/g prot in the normal control group, sham operation group, OJ group and study group, respectively. MDA content in the liver homogenate in the OJ model group was the highest followed by the study group, normal control group and sham operation group (Fig. 2). The difference in MDA content was statistically significant among the groups (P<0.05). MDA content in the study group was significantly reduced compared to that in the OJ model group, but was still higher than the content in the normal control group and sham operation group (P<0.05).

### Activity levels of SOD, GSH-Px and CAT in the liver homogenate

**Figure 3:**
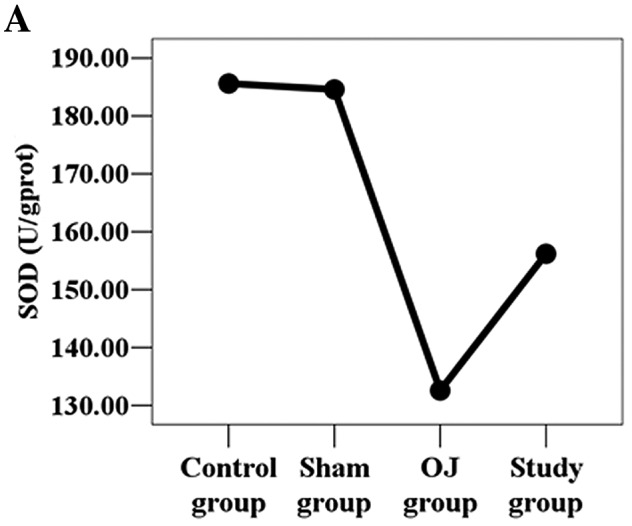

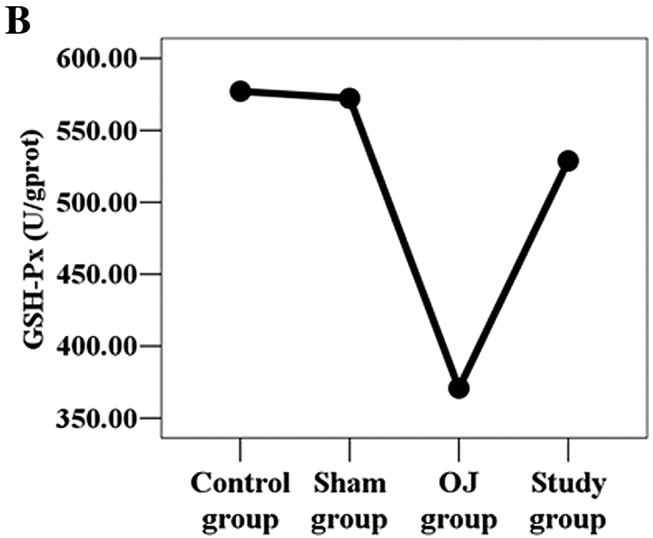

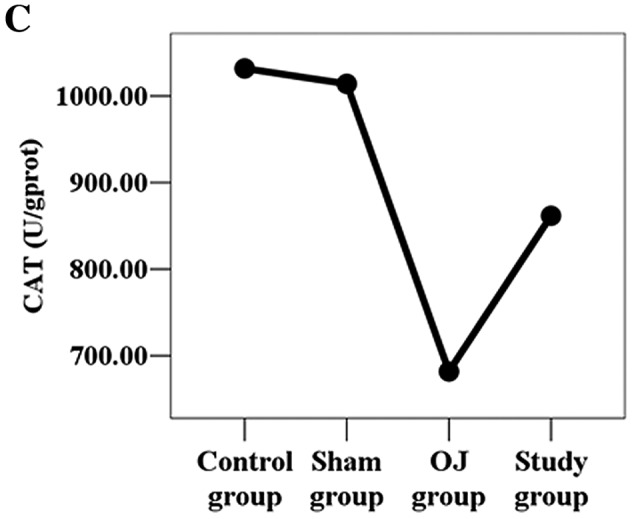
Changes in (A) SOD, (B) GSH-Px and (C) CAT activity in the liver homogenate among the groups.

The activity of SOD, GSH-Px and CAT in the liver homogenate in the normal control, sham operation, OJ and study groups, respectively, were 185.61±2.82, 184.59±2.75, 132.56±11.51, 156.18±14.79 U/g prot (SOD); 577.12±9.21, 572.25±7.05, 370.74±18.37, 528.76±18.76 U/g prot (GSH-Px); 1031.80±64.08, 1014.07±64.65, 681.81±125.02, 861.66±32.48 U/g prot (CAT). Among the groups, the difference in activity of SOD, GSH-Px and CAT in the liver homogenate was statistically significant (P<0.05). The activity levels of SOD, GSH-Px and CAT were lowest in the OJ model group. The study group had higher activities that the OJ group (P<0.05), and the normal control group and sham operation group had the highest activities (Fig. 3).

## Discussion

Obstructive jaundice (OJ) is a jaundice caused by mechanical obstruction of the bile duct caused by congenital malformations, inflammation, stones, tumors, parasitic disease and cholestasis. Its main syndromes include tissue damage to the liver and gallbladder, and a series of pathological and physiological changes in various body systems. Furthermore, it can cause dysfunction of intestinal mucous membrane, sepsis and multiple organ failure (10).

In recent years, numerous studies concerning the mechanisms of liver injury in OJ have been carried out and reveal that reactive oxygen species (ROS) play an important role. Oxygen radicals can peroxidize unsaturated fatty acids in biomembranes to form lipid hydroxide (LPO). LPO can reduce membrane fluidity, destroy membrane integrity, increase membrane permeability, and cause cell swelling and increased liver volume (11). MDA is one of the end-products of lipid peroxidation and can be used as an index of lipid peroxidation (12). Normal cells have a complex anti-ROS defense system that includes enzyme-catalyzed and non-enzymatic methods to reduce active oxygen radicals. Antioxidant enzymes include SOD, GSH-Px and CAT. Non-enzymatic antioxidants include cellulose, amino acids and metalloproteins. Cells produce small amounts of active oxygen radicals under normal physiological states that can be removed by intracellular antioxidant enzymes. SOD acts upon the superoxide anion to generate hydrogen peroxide which can be subsequently broken down into H_2_O and O_2_ by CAT and GSH-Px (13,14). This sequence reduces potential damage from hydrogen peroxide and prevents hydrogen peroxide from generating more harmful radicals (such as the hydroxide radical and alkoxy) through its interactions with O_2_ (9).

Capillary artemisia is the dry, ground part of *Artemisia scoparia* Waldst and Kit or *Artemisia capillaris* or feverfew. It is harvested when its spring seedlings are 6–10 cm high or when the autumn buds are growing. The stems are removed before drying. The products harvested during spring are usually called capillary wormwood, and those harvested in autumn are called *Artemisia capillaris* (15). Capillary artemisia is bitter, acrid and slightly cold, and moves across channels of the spleen, stomach, liver and gallbladder. Modern pharmacological studies have found that capillary artemisia has the following functions: it relaxes the gallbladder, protects the liver, reduces blood sugar, has antioxidant action and removes heat, dampness and jaundice. It is widely used in the clinical treatment of jaundice, hepatitis and other diseases (16).

In the present study, caapillary artemisia polysaccharide reduced liver weight, liver coefficient and MDA content as well as increased the activity levels of SOD, GSH-Px and CAT. These results indicate outstanding antioxidant and liver-protective effects, and suggest that these antioxidant effects of capillary artemisia may be a principal mechanism of effect. The results were consistent with our hypothesis. Our findings provide a theoretical basis for the clinical treatment of OJ using capillary artemisia polysaccharide.
